# Report of a Case of Pneumonia, to Atlanta Medical Society

**Published:** 1861-01

**Authors:** 


### ARTICLE II.

### *Report of a case of Pneumonia, to Atlanta Medical Society.*

[At a recent meeting of the Atlanta Medical Society, a resolution was passed, requesting members to report cases in writing as often as found convenient to do so. In compliance with that arrangement, at the last weekly meeting Dr. Hayden Coe reported the following case of Pneumonia, not on account of anything peculiar in it, but in order to present his views of the treatment applicable to the disease in its dynamic and adynamic forms. He went on to say, in connection with his written report, and in explanation of his object in making it, that whenever in Pneumonia the pulse is full and not very frequent blood-letting is indicated, and is preferable to other sedatives, such as veratrum, &c.; but when the pulse is frequent, say 120 to the minute, and small, bleeding, in his hands, has almost invariably proved injurious, and in this condition of the system veratrum is the proper sedative. Under such circumstances the action of this article lessens the number of beats, and increases the volume.—W. C. Moore, M. D., Secretary *pro tem.*]

Called to see Miss W——, æt. 16 or 17 years, on the 2d December inst., 24 hours after being attacked with a chill, soon followed by fever, which had continued up to the time of my visit, and was accompanied with pain in the right side. She had cough with bloody expectoration, flushed face; furred tongue; dry skin, but little above the the natural temperature; hurried respiration; and a quick, small pulse, numbering 125 beats to the minute. Prescribed 4 drops Tinct. Veratrum, in Syrup Squills, every three hours, and Calomel and Dovers' powder, as follows:

℞. Mild Chloride Mercury, grs. xii,
Pulvis Doveri, grs. xxxii.
Misce et fiat Chart. No. iv.

One to be given every five hours. Foot-bath, decoction of asclepiastu-berosa, and sinapism to the side at night.—Directed that the veratrum be suspended, should vomiting occur.

December 3d.—Had one or two slight discharges from the bowels since last visit—pulse 150, and larger—pain continues in the side. Ordered a blister to the right side and continued the veratrum, Dovers' powder and Calomel in the same doses.

December 4th.—12 o'clock, M., found her fully under the influence of veratrum, and had vomited several times during the forenoon, and suffered from constant nausea, and difficulty of breathing from spasm of the gottis. Pulse reduced to 60, and probably had been lower, from the report of the nurse. The unnecessary impression had been made on account of the failure to suspend the sedative when vomiting commenced, as had been directed. Pain has subsided, cheeks pale, and the patient complains of a sense of extreme prostration—has had slight alvine evacuations from the bowels—expectoration easy with very little blood—gave 15 drops of black-drop, which dissipated all the unpleasant effects of the veratrum in twenty or thirty minutes. Ordered expectorant mixture every three hours—Dovers' powder and Calomel at night, and also the foot-bath—veratrum to be commenced again, should the pulse rise above 100.

December 5th.—Patient decidedly convalescent—has had only one dose of veratrum since last visit, and has continued to improve rapidly. Pulse dose not, any time of day, exceed 100—has had one free evacuation from the bowels. Ordered at last visit expectorant, with Dovers' powder at night.

