# Errata

**Published:** 1861-02

**Authors:** 


### Errata.

In the case reported by Dr. H. Coe to the Atlanta Medical Society, and published in the January No. of our Journal, a typographical error occurs which destroys the sense of the article, and being a mistake in figures cannot be readily corrected by the reader. It occurs on page 264, 13th line from top. For 150 read 120.

